# Renal Transplantation: What Has Changed in Recent Years

**DOI:** 10.1155/2019/3618104

**Published:** 2019-06-20

**Authors:** Michele Santangelo, Lucrezia Furian, Nicos Kessaris, Karine Hadaya, Diederik Kimenai, Maria Irene Bellini

**Affiliations:** ^1^General Surgery and Kidney Transplantation Unit, “Federico II” University Hospital, Naples, Italy; ^2^Kidney and Pancreas Transplantation Unit, Padua University Hospital, Padua, Italy; ^3^Department of Nephrology and Transplantation, Guy's Hospital, Guy's and St Thomas' NHS Foundation Trust, London, UK; ^4^Department of Surgery, Nephrology and Transplantation Divisions, Geneva University Hospitals, Switzerland; ^5^Division of HPB & Transplant Surgery, Erasmus MC, University Medical Center Rotterdam, Rotterdam, Netherlands; ^6^Renal Transplant Directorate, Hammersmith Hospital, Du Cane Road, Imperial College NHS Trust, London, UK

Kidney transplantation is the best approved renal replacement therapy, although one of its biggest limitations remains a general organ donor shortage [1], not only in terms of absolute numbers, but particularly regarding preservation techniques.

The concept of static cold storage has been proved to damage marginal organs that are increasingly being accepted to match the waitlist demand. Modern dynamic preservation technologies have developed in recent years not only to actively prevent kidney damage before transplantation, but also to assess potential organ viability. In this special issue, containing 12 manuscripts, we focus on hypothermic machine perfusion, showing in a matched observational study by M. I. Bellini et al. a higher eGFR at one-year follow-up with the dynamic preservation compared to static cold storage. Moreover, during hypothermic machine perfusion, resistive index predicted delayed graft function (DGF) with accuracy of 0.78 and 0.87 for organs from donation after circulatory death (DCD) or after brain death (DBD), respectively, and significantly decreased incidence of DGF in DCD organs.

Another possible way to improve the standard preservation technique is described by A. Ostróżka-Cieślik et al., who found that the addition of ascorbic acid and prolactin to Biolasol solution affects the maintenance of the normal cytoskeleton of the stored graft. Furthermore, R. Thuillier and T. Hauet focused on the cytoskeleton integrity during cold preservation, highlighting the negative impact of hypothermia during static cold storage.

The existing barriers in transplantation are extensively reviewed by C. Steichen et al., in order to identify the development of novel strategies, such as machine perfusion and reliable biomarkers to monitor graft quality and predict short and long-term outcomes. To this end, it is noteworthy that severe injury of the renal microvasculature with relatively preserved tubular epithelium may be associated with some conditions of deceased kidney donors leading to early kidney graft nonfunction and loss as interestingly reported by N. Kojc et al., although not all the units rely on the use of histology preimplantation [2]. Long-term outcomes are in fact also related to other important conditions, and beyond rejection and delayed graft function, an accurate surveillance of immunosuppression should be constant to prevent side effects as malignancy, unfortunately more common in transplanted patients [3].

A. Reznik et al. describe the damage caused by ischemia reperfusion injury (IRI) in animal models. The group investigates the effects on AP-1 and Heat Shock Response in donor kidney parenchyma after warm ischemia, with the aim to map IRI related molecules as targets for reconditioning during machine perfusion.

In order to combat organ shortage and following the successful DCD programmes in the UK, Netherlands, and Spain, Poland has recently commenced its own DCD transplant series. The article from H. Stadnik et al. reports the preliminary evaluation of the first 19 such cases in the country, focusing on clinical outcomes as well as public perception as an essential step for the initiation and maintenance of the programme.

Another possible way to increase the organ donor pool is the utilisation of HCV positive donors. The use of the new direct-acting antiviral agents, whose safety and efficacy in eradicating HCV infection without an increased risk of allograft rejection, is analysed in the report by A. Calogero et al. The effects of HCV eradication, in terms of quality of life, are further elucidated by Sabbatini's trial, showing a meaningful improvement as a patient reported outcome in kidney transplant recipients.

The special issue also explores, through a systematic review, the potential benefits of living donors having laparoscopic versus robot assisted donor nephrectomy. The rate of postoperative pain in the latter group was significantly lower, confirming the safety and feasibility of this technique.

Finally, as dialysis is still the only available treatment for those who do not get transplanted, particular attention should be devoted to preserve their dialysis access. G. Ietto et al. present a retrospective analysis of patients who underwent native polycystic kidney nephrectomy and whether they can go back to peritoneal dialysis after surgery. Their findings suggest that those patients who presented with postoperative complications [4], such as bleeding and therefore potentially requiring reintervention, did not recover their peritoneal dialysis routine.

In conclusion, we believe that this special issue provides a valuable update on the scientific progress of renal transplantation, notably by adding insight and giving future direction on scientific research and clinical practice.

## References

[1] Bellini M. I., Koutroutsos K., Galliford J., Herbert P. E. (2017). One-year outcomes of a cohort of renal transplant patients related to BMI in a steroid-sparing regimen. *Transplantation Direct*.

[2] Santangelo M., Zuccaro M., de Rosa P. (2007). Older kidneys donor transplantation: five years' experience without biopsy and using clinical laboratory and macroscopic anatomy evaluation. *Transplantation Proceedings*.

[3] Bellini M. I., Gopal J. P., Hill P., Nicol D., Gibbons N. (2019). Urothelial carcinoma arising from the transplanted kidney: a single‐center experience and literature review. *Clinical Transplantation*.

[4] Santangelo M., Clemente M., Spiezia S. (2009). Wound complications after kidney transplantation in nondiabetic patients. *Transplantation Proceedings*.

